# Effects of tongue strengthening exercises in healthy adults and elderly: an integrative literature review

**DOI:** 10.1590/2317-1782/20232021213en

**Published:** 2023-06-05

**Authors:** Juliana Oliveira Silva, Lucia Dantas Giglio, Luciana Vitaliano Voi Trawitzki

**Affiliations:** 1 Departamento de Oftalmologia, Otorrinolaringologia e Cirurgia de Cabeça e Pescoço, Faculdade de Medicina de Ribeirão Preto - FMRP, Universidade de São Paulo - USP - Ribeirão Preto (SP), Brasil.

**Keywords:** Tongue, Pressure, Exercise, Adult, Aged

## Abstract

**Purpose:**

To summarize and discuss the scientific literature on the effects of tongue strengthening exercises on healthy adults and elderly people.

**Research strategies:**

We searched two online databases*,* PubMed and Web of Science.

**Selection criteria:**

Studies with evidence of interventions in tongues strengthening exercises in healthy individuals over 18 years of age.

**Data analysis:**

Study objectives, design, participants, interventions, gain in the percentage of tongue strength.

**Results:**

Sixteen studies were included. There was an increase in tongue strength after strengthening training in healthy adults and elderly people. This strength was maintained after a short period of detraining. We could not compare the results between age groups due to the different methodological designs. We found that the approach of a less intense training protocol was more effective in gaining tongue strength in the elderly.

**Conclusion:**

Tongue strength training proved effective in increasing tongue strength in healthy individuals of different age groups. The benefits reported for the elderly corresponded to the reversal of the progressive loss of strength and muscle mass caused by aging. These findings must be interpreted with caution considering the number of studies on the elderly and their methodological variability.

## INTRODUCTION

Tongue strength and resistance exercises are used in speech-language pathology clinical practice to recover the functional performance of the tongue. Muscle strength training is known to increase the volume, maximum isometric muscle strength, and neural activation of muscles^(1,2)^.

Like others used in clinical rehabilitation, these exercises are based on exercise physiology principles: intensity, concerning the amount of resistive load used in the exercises and their adequate frequency (number of repetitions and sets) and duration, aimed at generating better results and maintaining them for a longer period^(3)^. However, the most appropriate intensity to be used in tongue strength and resistance exercises is still not clear, especially for the elderly, whose conditions are already declined, being more susceptible to fatigue. Consequently, the intensity used in the exercises and their effects may differ between adults and the elderly.

Tongue strengthening exercises contribute to increasing the strength and thickness of the muscle and have a positive impact on swallowing, in addition to preventing sarcopenia, thus avoiding possible functional changes^(1,2)^. Sarcopenia is a condition of progressive loss of skeletal muscle mass and strength^(4)^ involving the tongue muscles^(5)^.

Training interruption is another aspect to be considered, that is, the period of detraining. Due to the limited duration of tongue muscle training, it is essential that the strength gained from the exercises remains after the training is complete.

Regarding the skeletal muscles, a few weeks after interrupting the strengthening training, the neural response to the muscle and its atrophy decreased, causing a lower maximum voluntary muscle strength acquired with the training^(6)^. However, studies still diverge regarding the strength decline in different detraining periods^(7,8)^.

In turn, age seems to influence changes in functional capacity after short- and long-term detraining. Toraman^(7)^ carried out a study whose elderly showed greater losses in functional performance from the training of body exercises than younger subjects.

The effects of detraining periods on the tongue muscle remain uncertain and there no studies have compared such a condition in different age groups.

Therefore, it is important to learn the strength and resistance parameters of the tongue for the elderly and their relationship with the proper functioning of swallowing to ensure efficient and safe swallowing and consequently better nutrition, hydration, and quality of life for this population.

## PURPOSE

This study aimed to summarize and discuss the scientific production addressing the effects of tongue-strengthening exercises on healthy adults and elderly people by observing the following aspects: (1) the best intensity for tongue strength exercises for healthy elderly individuals, (2) to analyze the percentage of strength gain after exercise for the elderly compared to healthy adults, and (3) to observe whether the elderly have a greater reduction in tongue strength after the detraining period than adults.

## RESEARCH STRATEGIES

We performed a literature search on two databases, PubMed and Web of Science, from the initial coverage period of each database until August 29, 2020. The descriptors were tongue OR lingual combined with strength OR force AND exercise OR training.

## SELECTION CRITERIA

Studies in English and/or Portuguese that met the following criteria were included: (1) evidence of an experimental study containing an intervention using isolated tongue strength exercises, (2) research involving healthy humans over 18 years of age, (3) objective values of tongue strength before and after exercises, and (4) use of a commercially available instrument to perform strength training and/or obtain objective measurements of tongue strength.

## DATA ANALYSIS

Initially, for the training carried out for the review three reviewers considered the abovementioned descriptors. A coincident number of articles found by each reviewer was considered an agreement.

One of the reviewers conducted an initial selection of articles based on the titles and abstracts, which were then analyzed by two other reviewers for purposes of relevance. As the initial selection, the same reviewer performed a detailed and careful analysis of the full texts and data extraction, such as study objective, design, participants, interventions, and main (objective measures of maximal tongue strength before and after training) and secondary goals (the training protocols, including the exercise intensity). Subsequently, another reviewer verified those data. Questions about screening and data extraction were resolved by consensus between the two reviewers.

We elaborated a table to standardize and select the following relevant data found in the articles: (1) origin and type of study; (2) sample size; (3) age of the participants; (4) exercise method; (5) instrument used to perform the training and measure tongue; (6) training intensity and duration; (7) percentage gain in tongue strength, and (8) percentage loss in tongue strength after the detraining period.

For items 7 and 8, basic arithmetic accounts were performed to convert objective numerical values into percentages.

## RESULTS

**Figure 1:**
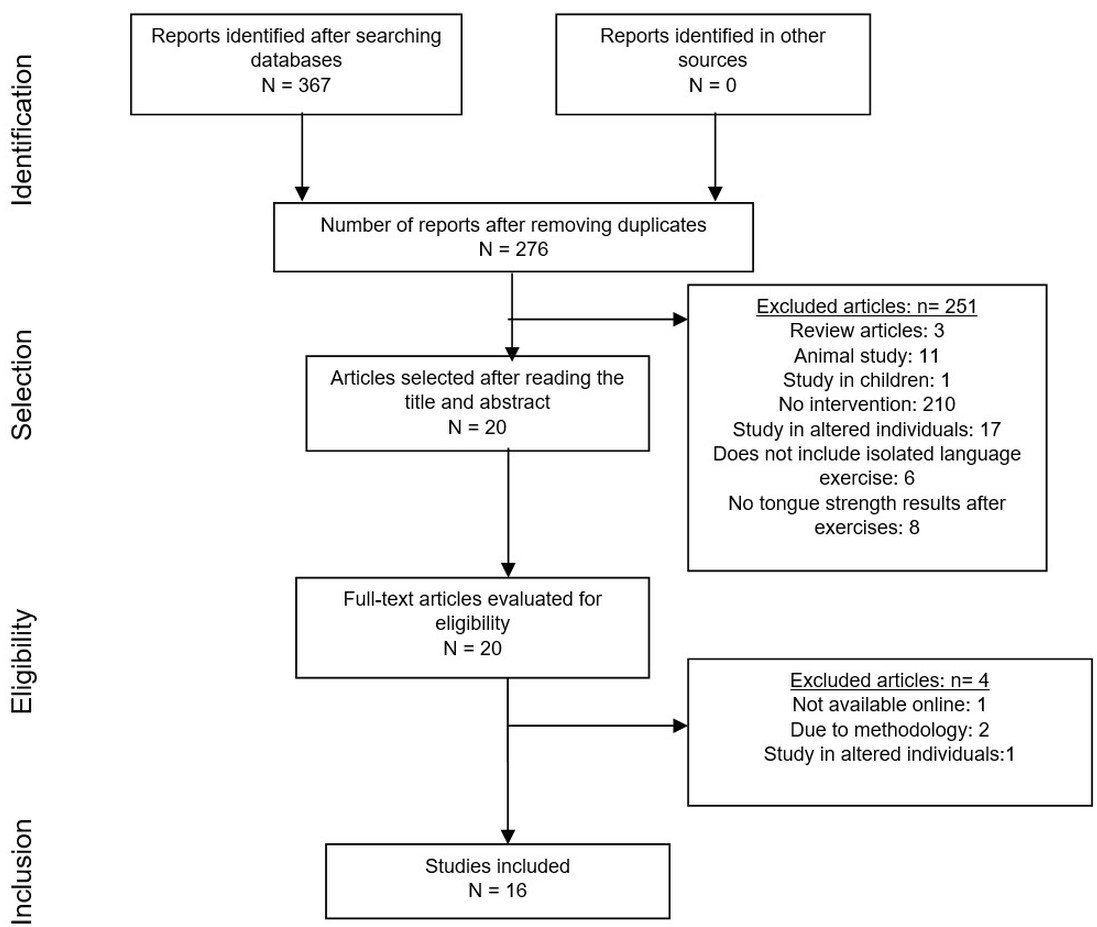
Study selection flowchart

Initially, 367 articles were found after searching the two databases. After excluding duplicate articles (91) and those that did not meet the eligibility criteria (259) or were not available in full text online (1), 16 articles remained, all in English, which were included in this study after reading in full. Figure 1 shows the flowchart based on PRISMA guidelines^(9)^.

### Characteristics of the studies

Out of the 16 studies selected for the review, 12 were randomized controlled trials and four were prospective cohort interventions. All studies reported on healthy individuals with no history of dysphagia, with 9 studies in adults^(10-16,17,18)^ and 7 with the elderly^(2,3,19-23)^ (Table 1). In all studies selected, at least one intervention group performed a tongue exercise, and only the data from these exercises were considered in this review.

**Table 1:** Characterization of studies with adults and elderly people

	Study	**N**	**Age**	**Instrument**
Adults	Lazarus et al.^(15)^ United States - Randomized clinical trial	G1=8; G2=10; GC=10	20 - 29	DL (G1) e IOPI (G2)
	Clark et al.^(16)^ United States - Randomized clinical trial	39	18-67 (average 37.8)	IOPI
	Clark^(14)^ United States - Randomized clinical trial	25	19-57 (average 29.8)	IOPI
	Arakawa et al.^(17)^ Japan - Randomized clinical trial	32	21 - 27	JMS
	Oh^(12)^ South Korea - Prospective cohort intervention	10	21-35	IOPI
	Yano et al.^(11)^ Japan - Prospective cohort intervention	11	20-21	JMS
	Hwang et al.^(10)^ - South Korea - Randomized clinical trial	30	20-28	TPS System and IOPI
	Lin et al.^(13)^ China - Randomized clinical trial	EG=44; GC=47	22-72 (average 35,3)	IOPI
	Park et al.^(18)^ South Korea - Randomized clinical trial	10	21-28	IOPI
Elderly	Robbins et al.^(1)^ - United States - Prospective cohort intervention	10	70 - 89	IOPI
	Van den Steen et al.^(19)^ - Belgium - Randomized clinical trial	G1=7; G2=9	70 - 95	IOPI
	Namiki et al.^(22)^ - Japan - Prospective cohort intervention	18	70 - 83	JMS
	Park et al.^(2)^ - South Korea - Randomized clinical trial	20	65 - 73	TPS System
	Van den Steen et al.^(20)^ - Belgium - Randomized clinical trial	G1=15; G2=16; G3=16	70-90	IOPI
	Lee et al.^(21)^ - South Korea - Randomized clinical trial	22	65 - 85	IOPI
	Szynkiewicz et al.^(23)^ - United States - Randomized clinical trial	G1=7; G2=8	60-86	IOPI

**Caption:** EG= Experimental Group; GC= Control Group; G1= Group 1; G2= Group 2; G3= Group 3; DL= Tongue Depressor; IOPI= Iowa Oral Performance Instrument (IOPI Medical, Redmond, WA); JMS= JMS tongue pressure measurement system (JMS Co. Ltd, Tokyo, Japan); TPS System=(TPS 100, CybermedicInc, Iksan, South Korea).

The studies used the following three different devices to collect the maximum lingual pressure data: the Iowa Oral Performance Instrument (IOPI Medical, Redmond, WA), the JMS tongue pressure measurement system (JMS Co. Ltd, Tokyo, Japan), and the TPS system (TPS 100, CybermedicInc, Iksan, South Korea)^(2,10)^. In all three devices, the pressure was recorded through tongue contact with an air-filled plastic bulb. This bulb is connected to a device that provides visual feedback of the pressure generated by the tongue, in kilopascals (kPa), through a digital reader. In the TPS system, the pressure sensor can be connected to a tablet via Bluetooth interface, allowing for a language training through games.

### Training protocols

Table 2 shows the training protocols used describing the exercises and their frequency, intensity, and duration.

**Table 2:** Training protocols in adults and elderly

	**Study**	**Exercises**	**Duration** **(weeks)**	**Frequency**	**Load**
					**(% de 1 MR)**
Adults	Lazarus et al.^(15)^	Press the tongue against a tongue depressor or the IOPI bulb for 2s in four directions: left, right, protrusion, and elevation.	4	10 repetitions in each direction, 5 times a day for 5 days a week	Maximum contraction.
	Clark et al.^(16)^	Elevation: press the tongue against the hard palate	9	Sequential training group: perform only the lifting exercise for 3 weeks, followed by 3 weeks of the protrusion exercise and another 3 weeks of the lateralization exercise. They completed 3 sets with 10 repetitions of each exercise, with the lateralization exercise performed 5 times to the right and 5 times to the left in each set.	Maximum contraction.
		Protrusion: push the tongue against a blade positioned between the upper and lower incisors and stabilized with the support of the fingers		Concurrent training group: a set of 10 repetitions of each exercise.	
		Lateralization: push the lateral region of the tongue against a blade positioned between the maxillary and mandibular premolars.		Both groups performed the exercises three times a day, every day.	
	Clark^(14)^	Strength: Press the anterior region of the tongue against the instrument bulb. Isotonic resistance: press the anterior region of the tongue against the instrument bulb several times.	4	3 sessions per week. Strength: 5 sets of 5 repetitions.	Strength: maximum contraction.
		Power: make the /t/ sound as fast as possible and press the instrument's bulb		Isotonic resistance: 5 sets. Power: 5 sets of 10 reps	Resistance: 75.
		Speed: repeat the /t/ sound several times and as fast as possible.		Speed: 5 sets of 10s	Power: 75.
	Arakawa et al.^(17)^	Press the apex of the tongue against the gingivobuccal fold and rotate the tongue every 2s to the right and then to the left.	12	20 rotations to the right and 20 to the left, once a day and every day of the week	-
	Oh^(12)^	Press the instrument bulb, positioned between the tongue and the hard palate, first with the anterior and then the posterior region of the tongue for 2s each.	8	3 sessions per week	1st week: 60.
				Lasting 30 min each. Rest of 10s between each repetition.	Following weeks: 80.
	Yano et al.^(11)^	Press the anterior region of the tongue against the hard palate.	8	30 repetitions, three times a day, 3 days a week.	1st week: 60.
					Following weeks: 80.
	Hwang et al.^(10)^	G1: through a “feeding game”, the participant pressed the tongue against the instrument bulb at different intensities depending on the game situation.	6	A 30min session 5 days a week	G2: 70.
		G2: press the tongue against the IOPI bulb positioned on the hard palate.			
	Lin et al.^(13)^	Press the instrument bulb with the anterior and then the posterior region of the tongue for 10s each.	8	5 days a week, with each session lasting 30min 30 repetitions of an exercise for the anterior and posterior regions, respectively.	-
Elderly	Robbins et al.^(1)^	Pressing the tongue against the instrument bulb positioned between the tongue and the hard palate	6	30 repetitions, 3 times a day 3 days a week	First week: 60.
					Following weeks: 80.
	Van den Steen et al.^(19)^	Press the anterior (G1) and posterior (G2) regions of the tongue against the bulb of the instrument positioned between the tongue and the hard palate	8	3 sessions per week	80
				24 sets of 5 reps, with 30s rest between each set	
	Namiki et al.^(22)^	Press the entire tongue against the hard palate as hard as possible for 10s	4	2 sets with 5 repetitions each, with a 10s rest period between each repetition, twice a day, every day of the week.	Maximum contraction
	Park et al.^(2)^	Isotonic exercise: pressing the tongue against the instrument bulb positioned between the tongue and the hard palate	-	Isotonic exercise: 3 sets of 30 repetitions per day	70
		Isometric exercise: press the tongue against the instrument bulb and keep the contraction for 30s		Isometric exercise: 3 sets of 30s each per day	
	Van den Steen et al.^(20)^	Press the anterior and then the posterior region of the tongue against the bulb of the instrument positioned between the tongue and the hard palate	8	3 sessions per week	G1:100.
				24 sets of 5 reps (12 sets of each region), with 30s rest between each set	G2: 80.
					G3:60.
	Lee et al.^(21)^	Press the tongue against the instrument bulb positioned between the tongue and the hard palate	8	30 times, 3 times a day 3 days a week	-
	Szynkiewicz et al.^(23)^	G1- Pressing the tongue as hard as possible against a tongue depressor, during elevation, protrusion, and lateralization	6	3 sessions per week	Maximum contraction
		G2- Exercise mentioned above combined with the mental practice of lingual exercises using motor images		10 repetitions in each direction (protrusion, elevation, left lateralization, and right lateralization) 3 times a day	

**Caption:** G1= Group 1; G2= Group 2; G3 = Group 3; IOPI= Iowa Oral Performance Instrument; s= seconds. min=minutes. % 1RM: 1-repeated maximum contraction

### Effect of exercises on tongue strength

The mean values of maximum initial tongue strength ranged from 49 to 66 kPa among adults and from 31 to 45 kPa among the elderly. After the training period, all studies observed a significant increase in maximal lingual strength, with mean values ranging between 52 and 80 kPa in adults and 34 to 55 kPa in the elderly.

As for strength gain, the values were presented in percentage (%) considering the final strength values (after training) subtracted by the initial value (before training) divided by 100 (Table 3).

**Table 3:** Tongue strength measurements after training in adults and older adults

	**Study**	**Strength measurements after training (%)**			
Adults	Lazarus et al.^(15)^	Tongue depressor: 12.8		IOPI: 14.2	
	Clark et al.^(16)^	Strength in elevation: 6	Strength in lateralization: 26.6		Strength in protrusion: 13.4
	Clark^(14)^	Strength exercise: 25.5	Isotonic resistance exercise: 11.3	Power exercise: 10.6	Speed exercise: 10.4
	Arakawa et al.^(17)^	Men: 18.5		Women: 31.8	
	Oh^(12)^	Anterior region of the tongue: 24.8		Posterior region of the tongue: 25.6	
	Yano et al.^(11)^	Anterior region of the tongue: 45.2		Posterior region of the tongue: 50	
	Hwang et al.^(10)^	G1: 6.5		G2: 2.9	
	Lin et al.^(13)^	Anterior region of the tongue: 3.5		Posterior region of the tongue: 10.4	
	Park et al.^(20)^	10,4			
Elderly	Robbins et al.^(1)^	19,5			
	Van den Steen et al.^(19)^	Anterior region of the tongue: G1 = 72.4; G2= 15.1		Posterior region of the tongue: G1 = 59.7; G2= 44.9	
	Namiki et al.^(22)^	8,9			
	Park et al.^(2)^	18			
	Van den Steen et al.^(20)^	Anterior region of the tongue: G1 = 61; G2 = 60.4; G3 = 51.8		Posterior region of the tongue: G1 = 74.5; G2 = 50.2; G3 = 53.3	
	Lee et al.^(21)^	Anterior region of the tongue = 11.9		Posterior region of the tongue = 8.78	
	Szynkiewicz et al.^(23)^	G1=9		G2=16.9	

**Caption:** G1= Group 1; G2= Group 2; G3= Group 3; IOPI= Iowa Oral Performance Instrument.

In studies involving adult subjects, the percentage of tongue strength gain ranged from 2.9% to 31.8%. Yano et al. ^(11)^ stood out by presenting an average percentage of strength gain in the anterior region of the tongue of 45.2% and of 50% in the posterior region after 8 weeks of training. Van den Steen et al.^(19)^ and Van den Steen et al.^(20)^ found the highest percentages of strength gain by observing the effects of a tongue strength training protocol for 8 weeks on healthy elderly individuals. They obtained an average gain of 72.4% and 74.5% in tongue strength, respectively. In turn, the other studies with elderly individuals showed a percentage of tongue strength gain between 8.7% and 19.5%.

Only Van den Steen et al.^(19)^ studied the effects of strengthening exercises in the anterior and posterior regions of the tongue, separately. They found that the group that performed only the exercise in the anterior region had a greater percentage of increase in tongue strength for the same region (72.4%) and a lower strength percentage for the posterior region (15.1%). The second group that exercised only the posterior region obtained a greater strength gain for the anterior tongue (59.7%) than the exercised region (44.9%).

Yano et al.^(11)^ and Lee et al.^(21)^ observed only the strengthening effects on the anterior region of the tongue in adults and found different results. The first study had the greatest increase in tongue strength for the posterior region (50%) and not for the anterior region, which was the exercised area (45.2%). The second study showed a greater gain for the exercised region (11.9%) and a lower gain for the posterior region (8.7%).

Three studies used tongue strengthening exercises for both regions and found a higher percentage of strength gain for the posterior region of the tongue^(12,13,20).^

Clark et al.^(14)^ studied adult individuals and found that other types of training for the tongue muscle involving no strength gain as the main target exerted some effect on the tongue. Isotonic resistance exercise generated an 11.3% increase in tongue strength, while power and speed exercise increased tongue strength by 10.6% and 10.4%, respectively. However, only the strength exercise generated the greatest results, with a percentage increase of 25.5% in tongue strength.

Only Van den Steen et al.^(20)^ investigated the effects of tongue strengthening exercises on different values of resistance load. They observed a greater gain in tongue strength (anterior region: 61%; posterior region: 74.5%) for the elderly group that performed the exercise at 100% 1MR after 8 weeks of training compared to the other two groups that performed the exercise at 80% 1RM (anterior region: 60.4%; posterior region: 50.2%) and 60% 1RM (anterior region: 51.8%; posterior: 53.3%).

Namiki et al.^(22)^ and Szynkiewicz et al.^(23)^ also used tongue strengthening exercises at maximum contraction in the training protocol for elderly individuals. However, strength gain was much lower than that found by Van den Steen et al.^(20)^. The former found an increase of 8.9% after four weeks of training, while the latter reported an increase in tongue strength of 9% after six weeks of training. In Van den Steen et al^(20)^, it was only four weeks after the training started that the strength gain occurred for the anterior and posterior regions of the tongue, 42.7% and 58.6%, respectively. In adults, after four weeks of exercise at maximum tongue contraction, Lazarus et al.^(15)^ observed an increase of 14.2% in the group that exercised with the IOPI and 12.8% in the group that used the tongue depressant.

The mental practice of physical exercises also proved an effective method of strengthening the tongue muscles. Szynkiewiczet al.^(23)^ studied elderly individuals and found that tongue strength increased by 5.6% when using this method alone. However, higher results (16.9%) were achieved by combining mental practice and physical exercise for tongue strengthening. Herein, physical exercise alone resulted in a 9% increase in tongue strength.

### Effects of detraining on tongue strength

Only five studies^(11,12,16,19,20)^ investigated the effects of detraining on tongue strength. Those involving adults^(11,12,16)^ had a detraining period ranging from 4 to 28 weeks, and those with elderly individuals^(19,20)^ encompassed 4 weeks (Table 4). Four of those found no significant loss of tongue strength^(11,12,19,20)^, while Clark et al.^(16)^ observed a significant reduction in strength four weeks after detraining.

**Table 4:** Tongue strength measurements after detraining in adults and older adults

	**Study**	**Detraining period**	**Strength measurements after training (%)**	
Adults	Lazarus et al.^(15)^	-	There were no detraining measures	
	Clark et al.^(16)^	4 weeks	Unable to get detraining values	
	Clark^(14)^	-	There were no detraining measures	
	Arakawa et al.^(17)^	-	There were no detraining measures	
	Oh^(12)^	28 weeks	Anterior region of the tongue: -8.3	Posterior region of the tongue: -9.7
	Yano et al.^(11)^	12 weeks	Anterior region: -2.6	Posterior region: +10
	Hwang et al.^(10)^	-	There were no detraining measures	
	Lin et al.^(13)^	-	There were no detraining measures	
	Park et al.^(18)^	-	There were no detraining measures	
Elderly	Robbins et al.^(1)^	-	There were no detraining measures	
	Van den Steen et al.^(19)^	4 weeks	Anterior tongue strength: G1 = -6.7, G2 = +10.8	Posterior tongue strength: G1 = -10.5, G2 = -5.1
	Namiki et al.^(22)^	-	There were no detraining measures	
	Park et al.^(2)^	-	There were no detraining measures	
	Van den Steen et al.^(20)^	4 weeks	Anterior region of the tongue: G1 = -4.3, G2 = -2.9, G3 = +4.4 Posterior region of the tongue: G1 = + 4.1, G2 = - 5.1, G3 = + 1.5	
	Lee et al.^(21)^	-	There were no detraining measures	
	Szynkiewicz et al.^(23)^	-	There were no detraining measures	

**Caption:** G1 = Group 1; G2 = Group 2; G3 = Group 3.

### Considerations and implications for clinical practice

This literature review aimed to summarize the effects of tongue strengthening training on healthy adult and elderly individuals and compare their results considering especially the elderly population data. After the database search and careful analysis of the studies, 16 articles were selected to be part of this review. Objective measures of maximal tongue strength before and after training were considered as results of greatest interest and were converted into percentages for better visualization and comparison among the studies. The training protocols, including intensity, frequency, duration of exercises, and levels of resistance load, were considered results of secondary interest.

The comparison of all values of tongue strength before training allowed finding other articles that showed a decrease in tongue strength throughout the aging process.

Such a decline in the tongue strength for elderly individuals is related to changes in the tongue muscle composition that occur with age. In this age group, the tongue muscle composition has a higher fat percentage, especially in the posterior region, compared to young individuals. Such an accumulation of adipose tissue on the tongue impairs muscle performance in elderly individuals, which may worsen when associated with sarcopenia^(24)^.

In all studies analyzed, the tongue training generated a significant strength increase. Two studies with elderly individuals following the same training protocol^(19,20)^ reported the highest percentage increase. All other studies with elderly individuals used different training protocols, some consisting of daily exercises or even more than once a day; however, none of these studies reached an increase percentage in tongue strength like the two abovementioned studies.

Although elderly individuals have a lower tongue strength, thus being more susceptible to fatigue, three studies used a training protocol at maximum resistive load. Van den Steen et al.^(20)^ observed that the elderly group that performed the exercises at a load of 100% 1MR managed to complete the entire protocol without any sensation of pain or fatigue; in addition to having the best results for strength increase compared to two other groups that performed the exercises with a lower load. Two other studies also performed the tongue strength exercise at maximum contraction without mentioning neither pain nor fatigue for elderly individuals^(22,23)^. Such a scenario may be related to the composition of the tongue intrinsic muscle, mostly involving muscle fibers resistant to fatigue, such as type I and II fibers, mainly found in the body region and the tongue base^(25)^. It is worth noting that all three training protocols studied used few exercise repetitions and considerable rest periods, which may also have contributed to minimizing the development of fatigue.

Although Van den Steen et al.^(20)^ found that exercises in 100% 1MR provided a greater increase in tongue strength for elderly individuals, it does not represent a statistical difference compared with the two groups that performed the exercises in 60% 1MR and 80% 1MR. Furthermore, practicing exercises at lower loads generated a better success rate, that is, the elderly were able to perform the exercise adequately in most of their attempts, unlike the elderly who performed the exercise at maximum resistance load, who had a lower performance during the exercise repetitions.

The results suggest that for good outcomes for healthy elderly individuals, performing the exercise at high intensity may be unnecessary, that is, at maximum levels of resistance loads or daily training sessions. Using a lower resistance load, few exercise repetitions during the series, and rest periods allow for a significant gain in tongue strength, in addition to greater comfort and better performance, stimulating the elderly individuals to complete the training.

However, Van den Steen et al.^(19)^ and Van den Steen et al.^(20)^ found no effects of this training protocol on the tongue functional performance, such as the swallowing function, like in other studies with elderly individuals. This will be discussed in this review later on. Therefore, studies investigating the possibility of less intense protocols causing a tongue functional improvement are very relevant. In addition, it is also important to analyze the relationship between a greater gain in tongue strength and a better performance in these functions.

Furthermore, even though two studies with older adults^(19,20)^ showed the highest percentages of strength increase after the training period compared to all other studies included in this review, it is not possible to determine that this age group presents greater gains in tongue strength compared to adults. The studies vary concerning the training protocol used, presenting different durations and frequencies of exercises, and it is not possible to compare them without the some bias. However, it cannot be determined that both age groups, despite being healthy individuals and presenting baseline values of tongue strength within the normal range for their age, still acquire significant gains after training to strengthen the tongue muscles.

The same applies to the effects of detraining. Only Clark et al.^(16)^ reported a considerable decrease in tongue strength after four weeks of detraining in healthy adults, and used two different exercises in its training protocol. In addition, it is worth noting that the training protocol used in the study was more intense, consisting of daily exercises for 9 weeks, while in the other studies, the exercises were performed only three times a week for 8 weeks. Therefore, all these factors must be considered to clarify their influence on the effects of detraining.

In addition, the exercises interruption period is another essential factor to be considered. In the elderly population, the two studies that investigated the effects of detraining observed only a short period of four weeks^(19,20)^, and although some intervention groups of these two studies demonstrated a greater reduction in tongue strength compared to studies in adults by Oh^(12)^ and Yano et al.^(11)^, the other groups showed an increase in tongue strength, even after the exercises were interrupted. Therefore, it would be interesting to observe these effects in a longer detraining period, as performed by Oh^(12)^, who observed 28 weeks after exercise interruption. Thus, it would be possible to determine the real effects of detraining and state whether the elderly have a greater reduction in tongue strength than adults and, therefore, should perform the exercises regularly to regain and maintain tongue strength for a longer period.

The tongue pressure exercise on the palate proved effective and widely used to increase tongue strength, as it is an easy exercise to perform and does not require much physical effort, making it convenient for the elderly.

Still, Szynkiewicz et al.^(23)^ showed that mental practice combined with physical tongue exercises seems to be promising mainly aiming to reduce muscle fatigue and improve strength performance without using sensory stimuli^(26)^. Mental practice is believed to induce neural adaptations at the cortical level that generate positive effects on the coordination and motor activation of the muscles. Such a scenario occurs in physical exercises where the first weeks of training generate changes primarily from alterations in the nervous system and not in the muscle structure per se^(3,26)^. Mental practice is very advantageous especially for the more debilitated elderly who experience greater difficulties when performing physical exercises of tongue strengthening, thus having lower gains. Thereby, increasing exercises through motor imagination would help improve the effects of tongue strength and limit the development of muscle fatigue in these individuals.

The tongue strengthening exercise also demonstrated other positive effects. In addition to increasing the organ strength, some studies observed that strength training causes a significant increase in the tongue volume of the elderly^(1,2)^, as well as in the suprahyoid muscle volume, as reported by Park et al.^(2)^. Such an effect of tongue training on another muscle can be explained by the anatomical relationship between these structures, that is, during the tongue elevation movement, when pressing the hard palate, the suprahyoid muscles are also contracted, causing the floor of the mouth and the hyoid bone to elevate^(27)^. Therefore, all these structures would be subject to some change during the tongue muscle training.

Namiki et al.^(22)^ found such a significant increase in the anterior and superior movement of the hyoid bone and in the opening of the upper esophageal sphincter after the tongue strengthening exercises. The increase in the former item is also related to a higher strength of the suprahyoid muscle since it also acts in opening the upper esophageal sphincter^(28)^.

All these mechanisms are essential for safe and effective swallowing since during the pharyngeal swallowing phase these actions must occur competently to avoid complications, such as food stasis or alteration in the airway protection mechanism.

Namiki et al.^(22)^ observed other positive effects on swallowing. Elderly individuals with signs of presbyphagia had a reduction in food residues in piriform sinuses and in penetration and aspiration scale scores. All these effects were attributed to a higher tongue strength and superior excursion of the hyoid bone. Robbins et al.^(1)^ also observed the effects of tongue strength exercise on the penetration and aspiration scale; however, the elderly in their study no longer had these conditions before performing the exercises. Therefore, changes in the scale were no longer expected.

Robbins et al.^(1)^ and Szynkiewicz et al.^(23)^ also reported an increase in swallowing strength after tongue muscle training. Such finding was correlated to the principle of neuroplasticity transfer, in which the increase in volume and tongue muscle mass after exercises provides a more qualified structure to perform an efficient swallowing movement, as well as changes in neuroplasticity associated with an improved oral motor function^(1)^.

Other effects of tongue strength exercises reported for the elderly were improvement in tongue motor skills in diadochokinesis tests^(22)^, increase in the tongue thickness^(1,2)^, significant increase in the thickness of the suprahyoid muscles^(2)^ , and higher salivary flow rate^(21)^.

Furthermore, Namiki et al.^(22)^ observed a significant increase in the anterior and superior movement of the hyoid bone when opening the upper esophageal sphincter, and a decrease in the pharyngeal transit time and the scale of proportion of residues in pyriform sinuses. Robbins et al.^(1)^ and Szynkiewicz et al.^(23)^ also observed an increase in maximum swallowing pressures.

However, based on all these data, it is possible that tongue strengthening exercises promote important benefits not only in this musculature but concomitantly in mastication and swallowing functions. In addition, it can also prevent or even reverse the progressive loss of strength and tongue muscle mass caused by aging.

Thereby, tongue strengthening exercises for speech-language pathology practice is even more clinically relevant, being essential for a speech-language pathology follow-up at this stage of life through guidance and monitoring of exercises for a better quality of life for the elderly population.

Finally, it is worth emphasizing that the data herein are based on healthy subjects; therefore, the effects of tongue strengthening exercises may differ in individuals with orofacial myofunctional alterations or due to some structural or neurological alteration.

## CONCLUSION

Overall, this literature review described the tongue strength training protocols and their effects on healthy adults and the elderly, focusing on the latter age group. We found positive evidence regarding the effects of training, such as higher tongue strength in all studies analyzed, as well as the maintenance of this strength after a short detraining period. A notable finding was the effectiveness of a less intense training protocol in providing greater results in tongue strength for the elderly, as well as the possibility of tongue strengthening exercises reversing the progressive loss of strength and muscle mass caused by aging. However, it is worth emphasizing that due to the small number of studies on the elderly and the methodological variability applied in the studies, our findings must be interpreted with caution.

## References

[1] Robbins J, Gangnon RE, Theis SM, Kays SA, Hewitt AL, Hind JA (2005). The effects of lingual exercise on swallowing in older adults. J Am Geriatr Soc.

[2] Park JS, Lee SH, Jung SH, Choi JB, Jung YJ (2019). Tongue strengthening exercise is effective in improving the oropharyngeal muscles associated with swallowing in community-dwelling older adults in South Korea: a randomized trial. Medicine.

[3] Burkhead LM, Sapienza CM, Rosenbek JC (2007). Strength-training exercise in dysphagia rehabilitation: principles, procedures, and directions for future research. Dysphagia.

[4] Cruz-Jentoft AJ, Baeyens JP, Bauer JM, Boirie Y, Cederholm T, Landi F (2010). Sarcopenia: European consensus on definition and diagnosis: report of the European working group on sarcopenia in older people. Age Ageing.

[5] Kobuchi R, Okuno K, Kusunoki T, Inoue T, Takahashi K (2020). The relationship between sarcopenia and oral sarcopenia in elderly people. J Oral Rehabil.

[6] Colliander EB, Tesch PA (1992). Effects of detraining following short term resistance training on eccentric and concentric muscle strength. Acta Physiol Scand.

[7] Toraman NF (2005). Short term and long term detraining: is there any difference between young-old and old people?. Br J Sports Med.

[8] Carvalho MJ, Marques E, Mota J (2009). Training and detraining effects on functional fitness after a multi component training in older women. Gerontology.

[9] Galvão TF, Pansani TSA, Harrad D (2015). Principais itens para relatar revisões sistemáticas e meta-análises: a recomendação PRISMA. Epidemiol Serv Saude.

[10] Hwang NK, Kim MJ, Lee G, Yoon T, Park JS, Jung Y (2020). Effect of tongue-strengthening training combined with a tablet personal computer game in healthy adults. J Oral Rehabil.

[11] Yano J, Yamamoto-Shimizu S, Yokoyama T, Kumakura I, Hanayama K, Tsubahara A (2019). Effects of anterior tongue strengthening exercises on posterior tongue strength in healthy young adults. Arch Oral Biol.

[12] Oh JC (2015). Effects of tongue strength training and detraining on tongue pressures in healthy adults. Dysphagia.

[13] Lin CH, Chung SY, Lin CT, Hwu YJ (2021). Effect of tongue-to-palate resistance training on tongue strength in healthy adults. Auris Nasus Larynx.

[14] Clark HM (2012). Specificity of training in the lingual musculature. J Speech Lang Hear Res.

[15] Lazarus C, Logemann JA, Huang CF, Rademaker AW (2003). Effects of two types of tongue strengthening exercises in young normals. Folia Phoniatr Logop.

[16] Clark HM, O’Brien K, Calleja A, Corrie SN (2009). Effects of directional exercise on lingual strength. J Speech Lang Hear Res.

[17] Arakawa I, Koide K, Takahashi M, Mizuhashi F (2015). Effect of the tongue rotation exercise training on the oral functions in normal adults - Part 1 investigation of tongue pressure and labial closure strength. J Oral Rehabil.

[18] Park JW, Hong HJ, Nam K (2020). Comparison of three exercises on increasing tongue strength in healthy young adults. Arch Oral Biol.

[19] Van den Steen L, Schellen C, Verstraelen K, Beeckman AS, Vanderwegen J, De Bodt M (2018). Tongue-strengthening exercises in healthy older adults: specificity of bulb position and detraining effects. Dysphagia.

[20] Van den Steen L, Vanderwegen J, Guns C, Elen R, De Bodt M, Van Nuffelen G (2019). Tongue-strengthening exercises in healthy older adults: does exercise load matter? A randomized controlled trial. Dysphagia.

[21] Lee KH, Jung ES, Choi YY (2020). Effects of lingual exercises on oral muscle strength and salivar flow rate in elderly adults: a randomized clinical trial. Geriatr Gerontol Int.

[22] Namiki C, Hara K, Tohara H, Kobayashi K, Chantaramanee A, Nakagawa K (2019). Tongue-pressure resistance training improves tongue and suprahyoid muscle functions simultaneously. Clin Interv Aging.

[23] Szynkiewicz SH, Kamarunas E, Drulia T, Nobriga CV, Griffin L, O’Donoghue CR (2021). A randomized controlled trial comparing physical and mental lingual exercise for healthy older adults. Dysphagia.

[24] Nakao Y, Yamashita T, Honda K, Katsuura T, Hama Y, Nakamura Y (2021). Association among age-related tongue muscle abnormality, tongue pressure, and presbyphagia: a 3D MRI Study. Dysphagia.

[25] Sanders I, Mu L, Amirali A, Su H, Sobotka S (2013). The human tongue slows down to speak: muscle fibers of the human tongue. Anat Rec.

[26] Slimani M, Tod D, Chaabene H, Miarka B, Chamari K (2016). Effects of mental imagery on muscular strength in healthy and patient participants: a systematic review. J Sports Sci Med.

[27] Hori K, Taniguchi H, Hayashi H, Magara J, Minagi Y, Li Q (2013). Role of tongue pressure production in oropharingeal swallow biomechanics. Physiol Rep.

[28] Jacob P, Kahrilas PJ, Logemann JA, Shah V, Ha T (1989). Upper esophageal sphincter opening and modulation during swallowing. Gastroenterology.

