# Supplementary material for: Effects of tongue strengthening exercises in healthy adults and elderly: an integrative literature review
Source: Codas. 2023 Jun 5;35(3):e20210213. doi: 10.1590/2317-1782/20232021213en (PMC10266797; doi:10.1590/2317-1782/20232021213en)
Supplement: Translation

# Translation: Efeitos dos exercícios de força da língua em adultos e idosos saudáveis: uma revisão integrativa de literatura

**DOI:** 10.1590/2317-1782/20232021213pt

**Authors:** Juliana Oliveira Silva, Lucia Dantas Giglio, Luciana Vitaliano Voi Trawitzki

**Affiliations:** 1 Departamento de Oftalmologia, Otorrinolaringologia e Cirurgia de Cabeça e Pescoço, Faculdade de Medicina de Ribeirão Preto - FMRP, Universidade de São Paulo - USP - Ribeirão Preto (SP), Brasil.

**Keywords:** Língua, Pressão, Exercício Físico, Adulto, Idoso

## Abstract

**Objetivo:**

resumir e discutir a produção científica dos efeitos dos exercícios de força da língua em adultos e idosos saudáveis.

**Estratégia de pesquisa:**

foi realizada em dois bancos de dados *online, PubMed e Web of Science.* **Critérios de seleção:** foram considerados estudos que obtiveram evidências de intervenções com exercícios de força de língua em indivíduos saudáveis e acima de 18 anos de idade.

**Análise dos dados:**

foram extraídos os dados objetivos do estudo, desenho, participantes, intervenções, assim como ganho em percentual da força de língua.

**Resultados:**

foram incluídos 16 estudos. Houve um aumento da força da língua após o treino de fortalecimento em adultos e idosos saudáveis. Houve a manutenção dessa força após um período curto de destreino. Não foi possível comparar os resultados entre as faixas etárias, devido aos diferentes desenhos metodológicos. Notou-se que a abordagem de um protocolo de treinamento menos intenso se mostrou mais eficaz no ganho de força da língua em idosos.

**Conclusão:**

Os treinamentos de força de língua demonstraram ser eficazes para o seu aumento em indivíduos saudáveis de diferentes faixas etárias. Os benefícios relatados aos idosos foram a reversão da perda progressiva de força e massa muscular causada pelo envelhecimento. Os achados devem ser interpretados com cautela, devido ao número de estudos em idosos e sua variabilidade metodológica.

## INTRODUÇÃO

Os exercícios de força e resistência da língua são utilizados na prática clínica fonoaudiológica a fim de recuperar o desempenho funcional da língua. Sabe-se que o treino de força muscular provoca o aumento do volume e da força muscular isométrica máxima, além de aumentar a ativação neural dos músculos^(1,2)^.

Esses exercícios, assim como outros usados na reabilitação clínica, são baseados em princípios da fisiologia do exercício, sendo um deles a intensidade, a qual abrange a quantidade de carga resistiva utilizada nos exercícios, como também a sua frequência (número de repetições e séries) e duração adequadas, a fim de gerar melhores resultados e mantê-los por um maior período de tempo^(3)^. Entretanto, ainda não é bem esclarecido a intensidade mais adequada a ser usada nos exercícios de força e resistência da língua, principalmente em idosos, que já possuem um declínio dessas condições e com isso estão mais suscetíveis à fadiga. Em razão disso, a intensidade utilizada nos exercícios pode se diferenciar entre os adultos e idosos, assim como os seus efeitos.

O exercício de fortalecimento da língua, além de contribuir para o aumento da força e espessura desse músculo, tem um impacto positivo na deglutição e pode contribuir na prevenção da sarcopenia, evitando possíveis alterações funcionais^(1,2)^. A sarcopenia é uma condição de perda progressiva da massa e força muscular esquelética^(4)^ que inclui os músculos da língua^(5)^.

Outro aspecto a ser levado em conta é a interrupção do treinamento, ou seja, o período de destreino. Visto que a duração do treinamento muscular da língua é limitado, é essencial que a força adquirida com os exercícios permaneça após o término do treinamento.

No que diz respeito a musculatura esquelética, após algumas semanas da interrupção do treinamento de força, há uma redução da resposta neural para o músculo e sua atrofia, causando uma redução na força muscular voluntária máxima adquirida com o treinamento^(6)^. Entretanto, ainda há uma divergência entre os estudos quanto ao início do declínio dessa força em diferentes períodos de destreino^(7,8)^.

No que se refere à idade, a mesma parece ter influência nas alterações da capacidade funcional após o destreino de curto e longo prazo. Os idosos do estudo de Toraman^(7)^ demonstraram maiores perdas no desempenho funcional, obtido com o treinamento de exercícios corporais, comparado aos sujeitos mais jovens.

Na musculatura da língua, os efeitos dos períodos de destreino permanecem incertos e ainda não há estudos que comparem essa condição em diferentes faixas etárias.

Portanto, é importante conhecer os parâmetros de força e resistência da língua em idosos e sua relação com o adequado funcionamento da deglutição, para garantir uma deglutição eficiente e segura, e consequentemente melhor nutrição, hidratação, e qualidade de vida à essa população.

## OBJETIVOS

Os objetivos deste estudo foram resumir e discutir a produção científica relativa aos efeitos dos exercícios de força da língua em adultos e idosos saudáveis e observar os seguintes aspectos: (1) a melhor intensidade a ser usada nos exercícios de força da língua em idosos saudáveis, (2) analisar o percentual de ganho de força após exercícios em idosos comparando aos adultos saudáveis e (3) observar se os idosos possuem maior redução da força da língua após o período de destreino do que os adultos.

## ESTRATÉGIA DE PESQUISA

Uma pesquisa bibliográfica foi realizada em dois bancos de dados: *PubMed* e *Web of Science* no período inicial que cada banco abrangia até o dia 29 de agosto de 2020. Os descritores foram *tongue* *OR* *lingual* em combinação com *strength* *OR* *force* *AND* *exercise* *OR* *training*.

## CRITÉRIOS DE SELEÇÃO

Foram incluídos os estudos na língua inglesa e/ou portuguesa que atendiam aos seguintes critérios: (1) evidências de estudo experimental que continha intervenção com exercícios de força de língua isolado (2) pesquisa em seres humanos saudáveis e acima de 18 anos de idade; (3) valores objetivos da força da língua antes e após exercícios; (4) uso de instrumento disponível comercialmente para realizar o treinamento de força e/ou obter as medidas objetivas de força da língua.

## ANÁLISE DOS DADOS

Inicialmente foi realizado um treinamento para a revisão, considerando os descritores mencionados acima, por três revisores, confirmando a concordância entre eles, pelo coincidente número de artigos encontrados por cada revisor.

A seleção inicial dos artigos foi realizada por um revisor, a partir dos títulos e resumos, e posteriormente, analisados por mais dois revisores pela relevância. Uma análise detalhada e criteriosa dos textos completos e extração de dados, como objetivo do estudo, desenho, participantes, intervenções e resultados principais (medidas objetivas de força máxima da língua antes e após o treinamento) e secundários (os protocolos de treinamento, incluindo a intensidade dos exercícios) foi realizada pelo mesmo revisor da seleção inicial e depois verificadas por mais um revisor. Dúvidas quanto à triagem e extração de dados foram resolvidas por consenso entre dois revisores.

Uma tabela foi desenvolvida para padronizar e selecionar os dados relevantes encontrados nos artigos. Os dados selecionados foram: (1) origem e tipo do estudo; (2) tamanho da amostra; (3) idade dos participantes; (4) método do exercício; (5) instrumento utilizado para realizar o treinamento e mensurar a força da língua; (6) intensidade do treinamento e sua duração; (7) percentual do ganho de força da língua e (8) percentual de perda de força da língua após o período de destreino.

Para os itens 7 e 8 foram realizadas contas aritméticas básicas para conversão dos valores numéricos objetivos em porcentagem.

## RESULTADOS

Foram encontrados inicialmente 367 artigos após as buscas nos dois bancos de dados. Após a exclusão dos artigos duplicados (91) e aqueles que não se encaixavam nos critérios de elegibilidade (259) ou não estavam disponíveis em texto completo de forma online (1), restaram 16 artigos, todos na língua inglesa, os quais foram incluídos neste estudo após leitura na íntegra. A Figura 1 mostra o fluxograma baseado nas diretrizes PRISMA^(9)^.

**Figura 1:** Fluxograma da seleção dos estudos

### Características dos estudos

Dos 16 estudos selecionados para a revisão, 12 eram ensaios clínicos randomizados e 4 eram intervenções de coorte prospectiva. Todos os estudos relataram sobre indivíduos saudáveis e sem histórico de disfagia, sendo 9 estudos em adultos^(10-16,17,18)^ e 7 com idosos^(2,3,19-23)^ (Tabela 1). Em todos os estudos selecionados, pelo menos um grupo de intervenção realizou exercício de língua, sendo somente os dados desses exercícios considerados nesta revisão.

**Tabela 1:** Caracterização dos estudos com adultos e idosos

	**Estudo**	**N**	**Idade**	**Instrumento**
Adultos	Lazarus et al.^(15)^ Estados Unidos - Ensaio clínico randomizado	G1=8; G2=10; GC=10	20 - 29	DL (G1) e IOPI (G2)
	Clark et al.^(16)^ Estados Unidos - Ensaio clínico randomizado	39	18-67 (média 37,8)	IOPI
	Clark^(14)^ Estados Unidos - Ensaio clínico randomizado	25	19-57 (média 29,8)	IOPI
	Arakawa et al.^(17)^ Japão - Ensaio clínico randomizado	32	21 - 27	JMS
	Oh^(12)^ Coréia do Sul - Intervenção de coorte prospectiva	10	21-35	IOPI
	Yano et al.^(11)^ Japão - Intervenção de coorte prospectiva	11	20-21	JMS
	Hwang et al.^(10)^ - Coréia do Sul - Ensaio clínico randomizado	30	20-28	Sistema TPS e IOPI
	Lin et al.^(13)^ China - Ensaio clínico randomizado	GE=44; GC=47	22-72 (média 35,3)	IOPI
	Park et al.^(18)^ - Coréia do Sul - Ensaio clínico randomizado	10	21-28	IOPI
Idosos	Robbins et al.^(1)^ - Estados Unidos - Intervenção de coorte prospectivo	10	70 - 89	IOPI
	Van den Steen et al.^(19)^ - Bélgica - Ensaio clínico randomizado	G1=7; G2=9	70 - 95	IOPI
	Namiki et al.^(22)^ - Japão - Intervenção de coorte prospectiva	18	70 - 83	JMS
	Park et al.^(2)^ - Coréia do Sul - Ensaio clínico randomizado	20	65 - 73	Sistema TPS
	Van den Steen et al.^(20)^ - Bélgica - Ensaio clínico randomizado	G1=15; G2=16; G3=16	70-90	IOPI
	Lee et al.^(21)^ - Coréia do Sul - Ensaio clínico randomizado	22	65 - 85	IOPI
	Szynkiewicz et al.^(23)^ - Estados Unidos - Ensaio clínico randomizado	G1=7; G2=8	60-86	IOPI

**Legenda:** GE= Grupo Experimental; GC= Grupo Control; G1= Grupo 1; G2= Grupo 2; G3= Grupo 3; DL= Depressor de língua; IOPI= *Iowa Oral Performance Instrument* (IOPI Medical, Redmond, WA); JMS=sistema de medida de pressão da língua JMS (JMS Co. Ltd, Tóquio, Japão); Sistema TPS=(TPS 100, CybermedicInc, Iksan, Coréia do Sul).

Os estudos utilizaram 3 equipamentos diferentes para coletar os dados da pressão lingual máxima sendo eles: o *Iowa Oral Performance Instrument* (IOPI Medical, Redmond, WA), o sistema de medida de pressão da língua JMS (JMS Co. Ltd, Tóquio, Japão) e o sistema TPS (TPS 100, CybermedicInc, Iksan, Coréia do Sul)^(2,10)^. Nos três dispositivos, a pressão foi registrada por meio do contato da língua com um bulbo de plástico preenchido por ar. Este bulbo é conectado a um dispositivo que fornece o feedback visual da pressão gerada pela língua, em kilopascal (kPa), por meio de um leitor digital. No sistema TPS, o sensor de pressão pode ser conectado a um *tablet* por meio da interface *Bluetooth,* permitindo o treinamento da língua por meio de jogos.

### Protocolos de treinamento

Os protocolos de treinamento utilizados podem ser vistos na Tabela 2 que apresenta a descrição dos exercícios utilizados bem como a frequência, intensidade e duração dos mesmos.

**Tabela 2:** Protocolos de treinamento em adultos e idosos

	**Estudo**	**Exercícios**	**Duração** **(semanas)**	**Frequência**	**Carga**
					**(% de 1 RM)**
Adultos	Lazarus et al.^(15)^	Pressionar a língua contra um depressor de língua ou o bulbo do IOPI durante 2s em quatro direções: esquerda, direita, protrusão e elevação.	4	10 repetições em cada direção, 5 vezes ao dia durante 5 dias na semana	Contração máxima
	Clark et al.^(16)^	Elevação: pressionar a língua contra o palato duro.	9	Grupo de treinamento sequencial: realizar apenas o exercício de elevação durante 3 semanas, seguidas de 3 semanas do exercício de protrusão e mais 3 semanas do exercício de lateralização. Completaram 3 séries com 10 repetições de cada exercício, sendo o exercício de lateralização realizado 5 vezes à direita e 5 vezes à esquerda em cada série.	Contração máxima
		Protrusão: empurrar a língua contra uma lâmina posicionada entre os incisivos superiores e inferiores e estabilizada com o apoio dos dedos.		Grupo de treinamento concomitante: um conjunto de 10 repetições de cada exercício.	
		Lateralização: empurrar a região lateral da língua contra uma lâmina posicionada entre os pré-molares superiores e inferiores.		Ambos os grupos realizaram os exercícios três vezes ao dia, todos os dias	
	Clark^(14)^	Força: pressionar a região anterior da língua contra o bulbo do instrumento	4	3 sessões por semana.	Força: contração máxima.
				Força: 5 séries de 5 repetições.	Resistência:75.
		Resistência isotônica: pressionar a região anterior da língua contra o bulbo do instrumento diversas vezes		Resistência isotônica: 5 séries.	Potência: 75.
		Potência: fazer o som /t/ o mais rápido possível e pressionando o bulbo do instrumento		Potência: 5 séries de 10 repetições	
		Velocidade: repetir o som /t/ diversas vezes e o mais rápido possível		Velocidade: 5 séries de 10s	
	Arakawa et al.^(17)^	Pressionar o ápice da língua contra a prega gengivobucal e realizar a rotação da língua a cada 2s para a direita e depois para a esquerda	12	20 rotações para a direita e 20 para a esquerda, uma vez ao dia e todos os dias da semana	-
	Oh^(12)^	Pressionar o bulbo do instrumento, posicionado entre a língua e o palato duro, primeiro com a região anterior e depois a posterior da língua durante 2s cada.	8	3 sessões por semana	1º semana: 60
				Com duração de 30 min cada. Descanso de 10s entre cada repetição.	Semanas seguintes: 80
	Yano et al. ^(11)^	Pressionar a região anterior da língua contra o palato duro	8	30 repetições, três vezes ao dia, 3 dias por semana.	1º semana: 60.
					Semanas seguintes: 80.
	Hwang et al.^(10)^	G1: através de um “Jogo de alimentação”, o participante pressionava a língua contra o bulbo do instrumento em diversas intensidades dependendo da situação do jogo.	6	Uma sessão de 30min em 5 dias da semana	G2: 70
		G2: pressionar a língua contra o bulbo do IOPI posicionado no palato duro.			
	Lin et al.^(13)^	Pressionar o bulbo do instrumento com a região anterior e depois a posterior da língua durante 10s cada.	8	5 dias na semana, com cada sessão durando 30min 30 repetições de exercício para as regiões anterior e posterior, respectivamente.	-
Idosos	Robbins et al.^(1)^	Pressionar a língua contra o bulbo do instrumento posicionado entre a língua e o palato duro	6	30 repetições, 3 vezes ao dia em 3 dias da semana	Primeira semana: 60.
					Semanas seguintes: 80.
	Van den Steen et al.^(19)^	Pressionar a região anterior (G1) e posterior (G2) da língua contra o bulbo do instrumento posicionado entre a língua e o palato duro	8	3 sessões por semana.	80
				24 séries de 5 repetições, com 30s de descanso entre cada série.	
	Namiki et al.^(22)^	Pressionar toda a língua contra o palato duro o mais forte possível durante 10s	4	2 séries com 5 repetições cada, com período de descanso de 10s entre cada repetição, duas vezes ao dia, todos os dias da semana.	Contração máxima.
	Park et al.^(2)^	Exercício isotônico: pressionar a língua contra o bulbo do instrumento posicionado entre a língua e o palato duro	-	Exercício isotônico: 3 séries de 30 repetições por dia.	70
		Exercício isométrico: pressionar a língua contra o bulbo do instrumento e manter a contração por 30s		Exercício isométrico: 3 séries de 30s cada por dia.	
	Van den Steen et al.^(20)^	Pressionar a região anterior e depois a posterior da língua contra o bulbo do instrumento posicionado entre a língua e o palato duro	8	3 sessões por semana	G1:100.
				24 séries de 5 repetições (12 séries de cada região), com 30s de descanso entre cada série.	G2: 80.
					G3:60.
	Lee et al.^(21)^	Pressionar a língua contra o bulbo do instrumento posicionado entre a língua e o palato duro	8	30 vezes, 3 vezes ao dia em 3 dias da semana	-
	Szynkiewic et al.^(23)^	G1- Pressionar a língua o mais forte possível contra um depressor de língua, durante a elevação, protrusão e lateralização	6	3 sessões por semana	Contração máxima.
		G2- Exercício citado anteriormente combinado com prática mental de exercícios linguais utilizando imagens motoras		10 repetições em cada direção (protrusão, elevação, lateralização esquerda e lateralização direita) 3vezes ao dia	

**Legenda:** G1= Grupo 1; G2= Grupo 2; G3 = Grupo 3; IOPI= *Iowa Oral Performance Instrument*; s= segundos; min=minutos. % 1RM: repetição única máxima

### Efeito dos exercícios na força da língua

Os valores médios de força máxima inicial da língua variaram de 49 a 66 kPa entre os adultos e 31 a 45 kPa entre os idosos. Após o período de treinamento, todos os estudos observaram um aumento significativo na força máxima lingual, com valores médios entre 52 a 80 kPa nos adultos e 34 a 55 kPa nos idosos.

Quanto ao ganho de força, os valores foram apresentados em porcentagem (%) considerando os valores de força finais (após o treino) subtraído pelo valor inicial (antes do treino) dividido por 100 (Tabela 3).

**Tabela 3:** Medidas de força da língua após o treinamento em adultos e idosos

	**Estudo**	**Medidas de força após o treinamento (%)**			
Adultos	Lazarus et al.^(15)^	Depressor de língua: 12,8		IOPI: 14,2	
	Clark et al.^(16)^	Força na elevação: 6	Força na lateralização: 26,6		Força na protrusão: 13,4
	Clark^(14)^	Exercício de força: 25,5	Exercício de resistência isotônica: 11,3	Exercício de potência: 10,6	Exercício de velocidade: 10,4
	Arakawa et al.^(17)^	Homens: 18,5		Mulheres: 31,8	
	Oh^(12)^	Região anterior da língua: 24,8		Região posterior da língua: 25,6	
	Yano et al.^(11)^	Região anterior da língua: 45,2		Região posterior da língua: 50	
	Hwang et al.^(10)^	G1: 6,5		G2: 2,9	
	Lin et al.^(13)^	Região anterior da língua: 3,5		Região posterior da língua: 10,4	
	Park et al.^(18)^	10,4			
Idosos	Robbins et al.^(1)^	19,5			
	Van den Steen et al.^(19)^	Região anterior da língua: G1 = 72,4; G2= 15,1		Região posterior da língua: G1 = 59,7; G2= 44,9	
	Namiki et al.^(22)^	8,9			
	Park et al.^(2)^	18			
	Van den Steen et al.^(20)^	Região anterior da língua: G1 = 61; G2 = 60,4; G3 = 51,8		Região posterior da língua: G1 = 74,5; G2 = 50,2; G3 = 53,3	
	Lee et al.^(21)^	Região anterior da língua = 11,9		Região posterior da língua = 8,78	
	Szynkiewicz et al.^(23)^	G1=9		G2=16,9	

**Legenda:** G1= Grupo 1; G2= Grupo 2; G3= Grupo 3; IOPI= *Iowa Oral Performance Instrument*.

Nos estudos com indivíduos adultos, o percentual de ganho de força da língua variou entre 2,9% a 31,8%. Yano et al.^(11)^, foi o estudo que mais se destacou, apresentando um percentual médio de ganho de força na região anterior da língua de 45,2% e na região posterior de 50% após 8 semanas de treinamento. Os estudos com maiores percentuais de ganho de força foram o de Van den Steen et al.^(19)^ e o Van den Steen et al.^(20)^, ambos observaram os efeitos de um protocolo de treinamento de força da língua durante 8 semanas em idosos saudáveis e obtiveram um ganho médio de 72,4% e 74,5% na força da língua, respectivamente. Enquanto os outros estudos com sujeitos idosos, apresentaram um percentual de ganho de força da língua entre 8,7% e 19,5%.

Van den Steen et al.^(19)^ foram os únicos que estudaram os efeitos dos exercícios de fortalecimento na região anterior e posterior da língua separadamente. Observou-se que o grupo que realizou apenas o exercício na região anterior, teve um maior percentual de aumento na força da língua nessa mesma região (72,4%) e um menor percentual na força da região posterior (15,1%), já o segundo grupo que exercitou apenas a região posterior, obteve um maior ganho na força da língua anterior (59,7%) comparado a região exercitada (44,9%).

Yano et al.^(11)^ e Lee et al.^(21)^ observaram apenas os efeitos do exercício de fortalecimento da região anterior da língua em adultos e ambos obtiveram resultados diferentes. No primeiro estudo citado o maior aumento da força da língua foi observado na sua região posterior (50%) e não na região exercitada, a anterior (45,2%). O segundo estudo apresentou um maior ganho na região exercitada (11,9%) e um ganho inferior na região posterior (8,7%).

Três estudos utilizaram um treinamento de força da língua exercitando ambas as regiões e foi observado um maior percentual de ganho de força na região posterior da língua^(12,13,20)^.

Clark et al.^(14)^ observaram em seu estudo, com adultos, que outros tipos de treinamento na musculatura lingual que não envolviam o ganho de força como alvo principal, provocou esse efeito na língua. O exercício de resistência isotônica gerou um aumento de 11,3% na força da língua, enquanto o exercício de potência e velocidade aumentou 10,6% e 10,4% a força da língua, respectivamente. Contudo, o próprio exercício de força foi o que gerou maiores resultados, apresentando um aumento percentual de 25,5% na força da língua.

Van den Steen et al.^(20)^ foi o único a investigar os efeitos dos exercícios de força da língua em diferentes valores de carga resistiva. Observaram-se que o grupo de idosos que realizou o exercício em 100% 1RM teve um maior ganho na força da língua (região anterior: 61%; região posterior: 74,5%) após as 8 semanas de treinamento comparado aos outros dois grupos que realizaram o exercício em 80% 1RM (região anterior: 60,4%; região posterior: 50,2%) e 60% 1RM (região anterior: 51,8%; região posterior: 53,3%).

Namiki et al.^(22)^ e Szynkiewicz et al.^(23)^ também utilizaram no seu protocolo de treinamento, os exercícios de força da língua em contração máxima com idosos, entretanto o ganho de força foi muito inferior comparado ao estudo de Van den Steen et al.^(20)^. No primeiro estudo citado, o aumento foi de 8,9% após 4 semanas de treinamento, enquanto no segundo, a força da língua teve um aumento semelhante de 9% após 6 semanas de treinamento. Em Van den Steen et al.^(20)^, logo após as quatro semanas do início do treinamento, a região anterior da língua apresentou um ganho de 42,7% na sua força e a região posterior de 58,6%. Em adultos, após 4 semanas realizando o exercício com contração máxima da língua, Lazarus et al.^(15)^, observaram um aumento de 14,2% no grupo que se exercitou com o IOPI e 12,8% no grupo que utilizou o depressor de língua.

A prática mental de exercícios físicos também demonstrou ser um método eficaz no fortalecimento da musculatura da língua. Szynkiewicz et al.^(23)^ observaram em seu estudo com idosos que ao utilizar apenas esse método a força da língua aumentou em 5,6%. Entretanto, ao combinar a prática mental com o exercício físico de fortalecimento da língua, os resultados foram maiores (16,9%). Neste estudo, o exercício físico isolado resultou em um aumento de 9% na força da língua.

### Efeitos do destreino na força da língua

Apenas cinco estudos^(11,12,16,19,20)^ investigaram os efeitos do destreino na força da língua. Nos estudos com adultos^(11,12,16)^ o período de destreino variou de 4 a 28 semanas e com idosos^(19,20)^ 4 semanas (Tabela 4). Em quatro deles não houve perda significativa na força da língua^(11,12,19,20)^, enquanto que no estudo de Clark et al.^(16)^ foi verificado uma redução significativa da força 4 semanas após o destreino.

**Tabela 4:** Medidas de força da língua após o destreino em adultos e idosos

	**Estudo**	**Período de destreino**	**Medidas de força após o destreino (%)**	
Adultos	Lazarus et al.^(15)^	-	Não houve medidas de destreino	
	Clark et al.^(16)^	4 semanas	Não foi possível obter os valores do destreino	
	Clark^(14)^	-	Não houve medidas de destreino	
	Arakawa et al.^(17)^	-	Não houve medidas de destreino	
	Oh^(12)^	28 semanas	Região anterior da língua: -8,3	Região posterior da língua: -9,7
	Yano et al.^(11)^	12 semanas	Região anterior: -2,6	Região posterior: +10
	Hwang et al.^(10)^	-	Não houve medidas de destreino	
	Lin et al.^(13)^	-	Não houve medidas de destreino	
	Park et al.^(18)^	-	Não houve medidas de destreino	
Idosos	Robbins et al.^(1)^	-	Não houve medidas de destreino	
	Van den Steen et al.^(19)^	4 semanas	Força da língua anterior: G1 = -6,7, G2 = +10,8	Força da língua posterior: G1 = -10,5, G2 = -5,1
	Namiki et al.^(22)^	-	Não houve medidas de destreino	
	Park et al.^(2)^	-	Não houve medidas de destreino	
	Van den Steen et al.^(20)^	4 semanas	Região anterior da língua: G1 = -4,3, G2 = -2,9, G3 = +4,4	Região posterior da língua: G1 = + 4,1, G2 = - 5,1, G3 = + 1,5
	Lee et al.^(21)^	-	Não houve medidas de destreino	
	Szynkiewicz et al.^(23)^	-	Não houve medidas de destreino	

**Legenda:** G1 = Grupo 1; G2 = Grupo 2; G3 = Grupo 3.

### Considerações e implicações para prática clínica

O objetivo desta revisão de literatura foi resumir os efeitos do treinamento de força lingual em adultos e idosos saudáveis e comparar os resultados nessas diferentes faixas etárias, considerando principalmente os dados da população idosa. Ao final da busca em bancos de dados e a análise criteriosa dos estudos, foram selecionados 16 artigos para inclusão nesta revisão. As medidas objetivas de força máxima da língua antes e após o treinamento foram consideradas como resultados de maior interesse e esses resultados foram convertidos em porcentagem para melhor visualização e comparação entre os estudos. Os protocolos de treinamento, no qual inclui a intensidade, como a frequência, duração dos exercícios e níveis de carga resistiva utilizados, foram considerados como resultados de interesse secundário.

Após comparar todos os valores de força da língua antes do treinamento, foi possível observar o que outros artigos já evidenciaram: a força da língua apresenta uma redução durante o processo de envelhecimento.

Esse declínio observado na força da língua em idosos está relacionado às mudanças na composição muscular da língua que ocorrem ao decorrer da idade. A musculatura lingual nessa faixa etária apresenta um maior percentual de gordura em sua composição, principalmente na região posterior, comparado a indivíduos jovens. Esse acúmulo de tecido adiposo na língua prejudica o desempenho da sua musculatura e em idosos essa condição pode se agravar ainda mais quando relacionada à sarcopenia^(24)^.

Após o período de treinamento da língua, foi observado um aumento significativo na sua força em todos os estudos analisados, o maior aumento percentual foi observado em dois estudos com idosos que seguiu o mesmo protocolo de treinamento^(19,20)^. Os outros estudos com idosos utilizaram diferentes protocolos de treinamento, sendo que alguns consistiam de exercícios diários ou até mesmo a realização do exercício mais de uma vez ao dia, entretanto nenhum desses estudos atingiu um percentual de aumento na força da língua semelhante aos dois estudos citados anteriormente.

Apesar dos idosos possuírem uma menor força da língua, estando assim mais suscetíveis à fadiga, três estudos utilizaram um protocolo de treinamento com carga resistiva máxima. Van den Steen et al.^(20)^ observaram que o grupo de idosos que realizou os exercícios em uma carga de 100% 1RM conseguiu completar todo o protocolo sem nenhuma sensação de dor ou fadiga e ainda apresentou os melhores resultados no aumento da força comparado aos outros dois grupos que realizou os exercícios com uma carga inferior. Outros dois estudos também realizaram o exercício de força da língua em contração máxima e não mencionaram nenhum relato de dor e fadiga nos idosos^(22,23)^. Este fato pode estar relacionado à composição da musculatura intrínseca da língua ser em sua maior parte por fibras musculares resistentes à fadiga, como as fibras tipo I e IIa, que são encontradas principalmente na região do corpo e base da língua^(25)^, ademais, é interessante notar que em todos os três estudos foram utilizados protocolos de treinamento com poucas repetições dos exercícios e períodos de descanso consideráveis, o que também pode ter contribuído na minimização do desenvolvimento de fadiga.

Apesar dos exercícios em 100% 1RM proporcionarem um maior aumento da força da língua nos idosos em Van den Steen et al.^(20)^, esse valor não apresentou diferença estatística dos outros dois grupos que realizaram os exercícios em 60% 1RM e 80% 1RM, e ainda, a prática dos exercícios nessas cargas inferiores exibiu uma melhor taxa de sucesso, ou seja, os idosos conseguiram realizar o exercício de forma adequada na maioria das suas tentativas, ao contrário dos idosos que realizaram o exercício em carga resistiva máxima, no qual apresentaram um menor desempenho durante as repetições dos exercícios.

Diante dos resultados apresentados, observa-se que possa ser desnecessária a realização do exercício em alta intensidade, ou seja, com níveis máximos de cargas resistivas ou sessões diárias de treinamento, para adquirir bons resultados em idosos saudáveis. A utilização de uma carga resistiva inferior, poucas repetições do exercício durante uma série e períodos de descanso, permitem um ganho significativo na força da língua e ainda provocam um maior conforto e melhor desempenho dos sujeitos durante a realização dos exercícios, deixando-os mais motivados para realizar todo o treinamento.

Entretanto, Van den Steen et al.^(19)^ e Van den Steen et al.^(20)^ não observaram os efeitos desse protocolo de treinamento no desempenho funcional da língua, como a função de deglutição, por exemplo, como foi realizado pelos outros estudos em idosos e que será discutido nesta revisão posteriormente. Portanto, torna-se notável a importância de estudos que investiguem a possibilidade desses protocolos menos intensos também provocarem uma melhora funcional da língua, como também, a relação entre um maior ganho de força da língua com um melhor desempenho nestas funções.

Ademais, mesmo dois estudos com idosos^(19,20)^ apresentando os maiores percentuais no aumento de força após o período de treinamento comparado a todos os outros estudos incluídos nesta revisão, não é possível determinar que essa faixa etária apresente maiores ganhos na força da língua comparado aos adultos. Os estudos variam muito em relação ao protocolo de treinamento utilizado, apresentando diferentes durações e frequências dos exercícios, não sendo possível compara-los sem a presença de um viés. Contudo, é possível determinar que ambas as faixas etárias, apesar de serem indivíduos saudáveis e apresentarem valores basais de força da língua dentro da faixa de normalidade para a sua idade, adquirem ainda, ganhos significativos após um treinamento de fortalecimento da musculatura lingual.

O mesmo se aplica aos efeitos do destreino. Apenas o estudo de Clark et al.^(16)^ relatou uma diminuição considerável na força da língua após 4 semanas de destreino em adultos saudáveis, e este foi o único estudo dentre os quatros citados anteriormente que utilizou em seu protocolo de treinamento mais dois exercícios diferentes, além disso, é importante notar que o protocolo de treinamento utilizado no estudo era mais intenso, consistindo de exercícios diários durante 9 semanas, enquanto os outros estudos, os exercícios eram realizados apenas três vezes por semana durante 8 semanas. Portanto, todos esses fatores devem ser considerados, a fim de esclarecer as suas influências nos efeitos do destreino.

Além disso, o período da interrupção dos exercícios é outro fator essencial a ser considerado. Na população idosa, os dois estudos que investigaram os efeitos de destreino, observaram apenas um período curto de 4 semanas^(19,20)^ e apesar de alguns grupos de intervenção desses dois estudos demonstrarem uma maior redução na força da língua comparado aos estudos em adultos de Oh^(12)^ e Yano et al.^(11)^, os demais grupos apresentaram aumento na força da língua, mesmo após a interrupção dos exercícios. Portanto, seria interessante observar esses efeitos em um maior período de destreino, como realizado no estudo de Oh^(12)^ que observou um período de 28 semanas após a interrupção dos exercícios. Desta forma, seria possível determinar os reais efeitos do destreino e afirmar se os idosos possuem uma maior redução na força da língua comparado aos adultos e, portanto, devem realizar os exercícios regularmente, a fim de recuperar e manter a força da língua por um maior período de tempo.

O exercício de pressão da língua no palato se mostrou eficaz e muito utilizado para o aumento da força lingual, por ser um exercício fácil de ser realizado e não exigir muito esforço físico, torna-se conveniente para os idosos.

Ainda, Szynkiewicz et al.^(23)^ mostrou que a prática mental combinada com exercícios físicos de língua parece ser promissora, sendo o seu principal objetivo reduzir a fadiga muscular e melhorar o desempenho da força sem o uso de estímulos sensoriais^(26)^. Acredita-se que a prática mental induz adaptações neurais em nível cortical que geram efeitos positivos na coordenação e ativação motora dos músculos, assim como é observado em exercícios físicos, em que nas primeiras semanas de treinamento as alterações provocadas por esse tipo de exercício são decorrentes primeiramente de modificações no sistema nervoso e não de mudanças na estrutura da musculatura em si^(3,26)^. A prática mental torna-se um fator muito vantajoso, principalmente para idosos mais debilitados que sentem uma maior dificuldade ao realizar os exercícios físicos de força da língua e com isso apresentam ganhos inferiores. Desse modo, o incremento de exercícios por meio da imaginação motora ajudaria a melhorar os efeitos da força da língua e ainda limitar o desenvolvimento da fadiga muscular nesses indivíduos.

O exercício de fortalecimento da língua ainda demonstrou outros efeitos positivos, além do aumento da força desse órgão, alguns estudos observaram que o treinamento de força provoca um aumento significativo no volume lingual de idosos^(1,2)^, como também no volume da musculatura supra-hióidea como relatado por Park et al.^(2)^. Esse efeito do treinamento da língua em uma outra musculatura pode ser explicado pela relação anatômica entre essas estruturas, ou seja, durante o movimento de elevação da língua, ao pressionar o palato duro, os músculos supra-hióideos também são contraídos, provocando a elevação do assoalho da boca e do osso hióide^(27)^. Portanto, todas essas estruturas estariam sujeitas a alguma mudança durante o treinamento da musculatura lingual.

Namiki et al.^(22)^ observaram esse aumento significativo na movimentação anterior e superior do osso hióide, como também um aumento na abertura do esfíncter esofágico superior após a realização dos exercícios de força da língua, o aumento neste último item também está relacionado ao aumento da força da musculatura supra-hióidea, já que a mesma também tem ação na abertura do esfíncter esofágico superior^(28)^.

Todos esses mecanismos são essenciais para uma deglutição segura e eficaz, visto que durante a fase faríngea da deglutição essas ações devem ocorrer de forma competente para não ocorrer complicações, como estase alimentar ou alteração no mecanismo de proteção das vias aéreas.

Outros efeitos positivos na deglutição também foram observados por Namiki et al.^(22)^. Os idosos que apresentavam sinais de presbifagia tiveram uma redução de resíduos alimentares em seios piriformes, assim como uma redução dos escores da escala de penetração e aspiração e todos esses efeitos foram atribuídos ao aumento da força da língua e da excursão superior do osso hióide. Robbins et al.^(1)^ também observaram os efeitos do exercício de força da língua sobre a escala de penetração e aspiração, entretanto, os idosos do seu estudo já não apresentavam essas condições antes da realização dos exercícios, portanto, mudanças na escala já não eram esperadas.

Um aumento na força de deglutição também foi relatada por Robbins et al.^(1)^ e Szynkiewicz et al.^(23)^ após o treinamento da musculatura lingual, este achado foi correlacionado ao princípio de transferência da neuroplasticidade, no qual o aumento do volume e massa muscular da língua após os exercícios fornece uma estrutura mais qualificada para realizar um movimento de deglutição eficiente, como também mudanças na neuroplasticidade associadas a melhora da função motora oral^(1)^.

Outros efeitos dos exercícios de força da língua relatados em idosos foram a melhora na habilidade motora da língua em provas de diadococinesia^(22)^, o aumento da espessura da língua^(1,2)^, o aumento significativo na espessura dos músculos supra-hioideos^(2)^ e o aumento da taxa do fluxo salivar^(21)^.

Ainda, Namiki et al.^(22)^ observaram em seu estudo que houve um aumento significativo na movimentação anterior e superior do osso hióide, na abertura do esfíncter esofágico superior e uma diminuição do tempo de trânsito faríngeo e na escala de proporção de resíduos em seios piriformes. Robbins et al.^(1)^ e Szynkiewicz et al.^(23)^ observaram também um aumento nas pressões máximas de deglutição.

Contudo, com bases em todos esses dados, é possível que os exercícios de fortalecimento da língua promovam benefícios importantes não somente nessa musculatura, mas concomitantemente nas funções de mastigação e deglutição, além disso, também possa prevenir ou até mesmo reverter a perda progressiva da força e massa muscular lingual provocada pelo envelhecimento.

Diante disso, a relevância clínica dos exercícios de força da língua para a prática fonoaudiológica torna-se ainda mais notável, sendo essencial um acompanhamento fonoaudiológico nessa etapa da vida, por meio de orientações e monitoramento dos exercícios, a fim de promover uma melhor qualidade de vida para a população idosa.

Por último, é importante enfatizar que os dados discutidos nesta revisão são provenientes de sujeitos saudáveis, portanto, os efeitos dos exercícios de força da língua podem ser diferentes em indivíduos com alterações miofuncionais orafaciais, seja decorrente de uma alteração estrutural ou neurológica.

## CONCLUSÃO

No geral, esta revisão de literatura descreveu os protocolos de treinamento de força da língua e seus efeitos em adultos e idosos saudáveis, tendo como foco esta última faixa etária. Evidências positivas quanto aos efeitos dos treinamentos foram encontradas, como o aumento da força de língua em todos os estudos analisados, assim como a manutenção dessa força após um período curto de destreino. Um achado notável, foi a eficácia de um protocolo de treinamento menos intenso fornecer maiores resultados na força da língua em idosos, como também, a possibilidade dos exercícios de fortalecimento da língua reverter a perda progressiva de força e massa muscular causada pelo envelhecimento. Entretanto, é importante ressaltar que devido ao pequeno número de estudos em idosos e pela variabilidade metodológica aplicada nos estudos, os achados devem ser interpretados com cautela.
